# Structural Stability Comparisons Between Natural and Engineered Group II Chaperonins: Are Crenarchaeal “Heat Shock” Proteins Also “pH Shock” Resistant?

**DOI:** 10.3390/microorganisms12112348

**Published:** 2024-11-18

**Authors:** Mercede Furr, Shadi A. Badiee, Sreenivasulu Basha, Shilpi Agrawal, Zeina Alraawi, Sobroney Heng, Carson Stacy, Yeasin Ahmed, Mahmoud Moradi, Thallapuranam K. S. Kumar, Ruben Michael Ceballos

**Affiliations:** 1 Department of Biology, University of Arkansas, Fayetteville, AR 72701, USA; mfurr@uark.edu (M.F.); sbasha@uark.edu (S.B.); 2 Department of Chemistry, University of Arkansas, Fayetteville, AR 72701, USA; sasadian@uark.edu (S.A.B.); sagrawal@uark.edu (S.A.); ziibrahe@uark.edu (Z.A.); moradi@uark.edu (M.M.); sthalla@uark.edu (T.K.S.K.); 3 Department of Molecular and Cell Biology, University of California Merced, Merced, CA 95343, USA; sobroneyheng@ucmerced.edu; 4 Cell and Molecular Biology Program, University of Arkansas, Fayetteville, AR 72701, USA; cstacy@uark.edu (C.S.); yahmed@uark.edu (Y.A.); 5 Quantitative Systems Biology Program, University of California Merced, Merced, CA 95343, USA

**Keywords:** group II chaperonin, Sulfolobales, thermotolerance, acido-tolerance, protein stability, heat shock protein, molecular dynamics, intrinsic fluorescence, circular dichroism, HSP60

## Abstract

Archaeal group II chaperonins, also known as heat shock proteins (HSPs), are abundantly expressed in Sulfolobales. HSPα and HSPβ gene expression is upregulated during thermal shock. HSPs form large 18-mer complexes that assist in folding nascent proteins and protecting resident proteins during thermal stress. Engineered HSPs have been designed for industrial applications. Since temperature flux in the geothermal habitats of Sulfolobales impacts intracellular temperature, it follows that HSPs have developed thermotolerance. However, despite the low pH (i.e., pH < 4) typical for these habitats, intracellular pH in Sulfolobales is maintained at ~6.5. Therefore, it is not presumed that HSPs have evolved acid-tolerance. To test tolerance to low pH, HSPs were studied at various pH and temperature values. Both circular dichroism and intrinsic fluorescence indicate that HSPα and HSPβ retain structural integrity at neutral pH over a wide range of temperatures. Structural integrity is compromised for all HSPs at ultra-low pH (e.g., pH 2). Secondary structures in HSPs are resilient under mildly acidic conditions (pH 4) but Anilino naphthalene 8-sulfonate binding shows shifts in tertiary structure at lower pH. Trypsin digestion shows that the HSPβ-coh backbone is the most flexible and HSPβ is the most resilient. Overall, results suggest that HSPα and HSPβ exhibit greater thermostability than HSPβ-coh and that there are limits to HSP acid-tolerance. Molecular dynamics (MD) simulations complement the wet lab data. Specifically, MD suggests that the HSPβ secondary structure is the most stable. Also, despite similarities in pH- and temperature-dependent behavior, there are clear differences in how each HSP subtype is perturbed.

## 1. Introduction

Species of the crenarchaeal family *Sulfolobaceae* (order: Sulfolobales) are among the most well-studied archaea [1,2,3,4,5,6,7,8,9,10,11,12,13,14,15]. Indeed, select archetypal strains (e.g., *S. solfataricus* P2) may be considered model systems [9,13,16,17,18,19]. These crenarchaea are known as hyperthermophiles for their ability to survive and thrive in acidic geothermal pools and volcanic hot springs at temperatures between 70 and 88 °C and pH < 4 [3,4,5,7,9]. One characteristic of these extremophiles that allows them to survive in what may be loosely described as “boiling battery acid” is a set of proteins known as heat shock proteins (HSP). 

HSPs are group II chaperonins that are found as three distinct subtypes within Sulfolobales: HSPα, HSPβ, and HSPγ [20,21,22,23,24,25,26]. More generally, these are called HSP60s since the molecular weight for each subtype is ~60 kDa [22,23,25,26,27]. However, it is HSPα and HSPβ that are commonly associated with thermal shock resistance, leading to the term “heat shock” proteins.

Upon incubation with adenosine triphosphate (ATP) and Mg^2+^ in vitro, HSP subunits form octadecameric (18-mer) complexes comprising two nonameric rings [21,22,28,29,30]. In nature, HSP complexes are reported to function in the folding of nascent proteins and in preventing aggregation and denaturing of other “client” proteins under physiological conditions and thermal stress, respectively [31,32,33,34]. HSP complexes form large (~1 MDa) molecular cages with stoichiometry favoring incorporation of HSPα and HSPβ [32,35,36,37]. Reports suggest that nascent, misfolded, or denatured client proteins associate with HSP complexes within the inner cavity of the cage structure. It is this association that stabilizes clients to ensure the proper folding of nascent protein or the re-folding of stressed protein [31,32,37,38]. It is also suggested that protein protection is accomplished by closing the chaperonin complex around all or part of the client, perhaps during complex formation or via client entry through an apical pore with an iris-like open-and-close function [39,40]. Whatever the mechanism, HSP complex–client protein associations perturb local energy minima in clients and appear to require ATP hydrolysis and divalent cations (e.g., Mg^2+^) [41,42]. In vitro, both homomeric and heteromeric 18-mer HSPα and HSPβ complexes will form in the presence of ATP and divalent cations [24,43]. Although several studies have reported in vitro HSP complex formation under conditions of thermal shock [20,21,22,23,36], until this study, it has not been conclusively demonstrated that HSP ring complexes form within the cell (see Figure S2). Furthermore, there is no detailed comparative analyses on the thermal stability of different HSP subtypes. Important to this study, it was noted in prior work from our lab that engineered HSP complexes designed to protect industrial enzymes significantly enhance enzymatic activity on sulfuric acid-treated substrates [24], prompting the question of whether HSPs are resilient when subjected to pH flux.

Despite surviving in acidic geothermal pools and volcanic hot springs with pH < 4, the intracellular environment of Sulfolobales is reported to be pH ~6.5 [44,45]. Therefore, there is no expectation that HSPs have evolved to be acid tolerant. In the present study, we employed biochemical and biophysical techniques to analyze the relative stability of natural HSPs (i.e., HSPα and HSPβ) and an engineered construct (i.e., HSPβ-coh) under different pH and temperature conditions to define the limits of acid- and thermotolerance of HSP subtypes. The wet lab data are complemented by extensive all-atom molecular dynamics (MD) simulations. Our results show differences in the thermal stability between HSP subtypes and limitations in the extent to which HSPs are acid tolerant.

## 2. Materials and Methods

### 2.1. Transmission Electron Microscopy

For visualizing HSP complexes via transmission electron microscopy (TEM), ~5 μL of protein suspension was spotted on a formvar-coated copper grid and incubated for 10 min in a humidity chamber. The grid was rinsed with distilled water and negatively stained with 2% (*w*/*v*) uranyl acetate for 2 min. The stain was wicked off and the sample was air-dried. Grids were imaged in a Hitachi H-7100 transmission electron microscope (Hitachi, Chicago, IL, USA) at 75 kV. Images were captured at 60,000–150,000× magnification. Double ring structures were resolvable at this magnification for HSPα and HSPβ.

### 2.2. Expression and Purification of Natural and Engineered Heat Shock Proteins

HSPα, HSPβ, and engineered HSPβ–coh fusion proteins were expressed and purified using previously described methods [24,43,46] with minor modifications. Briefly, natural HSPα and HSPβ from *S. shibatae* as well as the engineered HSPβ-coh were overexpressed via IPTG-induction in *E. coli* (i.e., BL21DE3-RIL codon+ cells; Invitrogen, Waltham, CA, USA). 

Cells were then pelleted via centrifugation at 7000 rpm for 15 min at 4 °C. Cell pellets were resuspended in buffer (50 mM Tris-HCl, 1 mM EDTA and pH 7.5) and then sonicated for 25 cycles (10 s on/off). The resulting lysate was centrifuged at 16,000× *g* for 20 min to separate the supernatant from cellular debris. The supernatant was heat-treated at 88 °C for 30 min and then centrifuged at 18,500× *g* for 30 min to remove heat labile proteins. Supernatant was then purified by Fast Perfusion Liquid Chromatography (FPLC) using an anion exchange column (Bio-Rad Q Sepharose Column, Hercules, CA, USA) with 150 mM to 1 M NaCl solution exchange and a flow rate of 3 mL/min. Eluted proteins were collected and spin-concentrated using a 30 kDa Amicon Ultra-15 spin concentration filter system (EMD Millipore, Burlington, MA, USA) followed by 16 h dialysis with 10 kDa SnakeSkin™ dialysis tubing (ThermoFisher Scientific, Waltham, MA, USA) at 4 °C in 20 mM Tris–HCl (pH 8.0). Purified proteins were stored at −80 °C for subsequent analyses. Eluted protein concentration was determined by the Bradford assay and analyzed using SDS PAGE.

### 2.3. Gel Electrophoresis and Silver Staining

Proteins were resolved using a 12% acrylamide resolving gel. All protein samples were mixed with SDS sample buffer [0.25% Coomassie Brilliant Blue (R250), 2% SDS, 10% glycerol (*v*/*v*), 100 mM tris, and 1% β-mercaptoethanol] at a ratio of 4:1 and boiled for 5 min. The boiled samples were loaded into respective wells alongside pre-stained protein molecular weight markers (ThermoFisher Scientific, Cat. No. 26620). Electrophoresis was performed at 150 V for 1 hr using the Bio-Rad Mini Protean protein gel electrophoresis system (BioRad, Hercules, CA, USA). Gels were stained with Coomassie Brilliant Blue and destained with a mixture of methanol, water, and acetic acid (4:4:1). After electrophoresis, gels were incubated in fixing solution [40% (*v*/*v*) methanol, 13.5% (*v*/*v*) formalin] for 10 min resulting in precipitation of protein and diffusion of SDS. Gels were then placed into an incubation solution (0.01 g Na_2_S_2_O_3_) for 1 min to oxidize protein. Gels were washed with water three times for 5 min and transferred into silver solution (i.e., 0.05 g silver nitrate) for another 10 min. Soluble proteins were visualized by replacing the silver solution with a secondary developing solution (1.5 g Na_2_CO_3_, 25 μL formalin, 50 μL of 0.02% Na_2_S_2_O_3_). The sodium carbonate in the latter solution reduces the silver nitrate attached to the proteins and thus the proteins adopt a brown color. As soon as the desired staining intensity was reached, the reaction was stopped by addition of citric acid solution (2.3 M), which was exchanged with water after 10 min.

### 2.4. Circular Dichroism (CD)

Far-UV circular dichroism (Far-UV CD) experiments were performed on a Jasco-1500 spectrophotometer (JASCO, Silver Spring, MD, USA). Thermal denaturation experiments were performed at temperatures ranging from 25 to 90 °C using 5 °C increments and protein concentrations of 0.3 mg/mL in 10 mM sodium phosphate buffer containing 10 mM NaCl with pH adjusted to 2, 4 and 7. The data were smoothened by using the Savitzky–Golay algorithm. For each temperature by pH condition, the Weighted Spectral Difference (WSD) was calculated [47]. The equation used to find the magnitude-weighted Euclidean distance between spectra was

$$WSD=\sqrt{\sum_{i=1}^{n}\left[\left(\frac{\left|y_{Ai}\right|}{n\cdot {\left|y_{A}\right|}_{avg}}\right){\left(y_{Ai}-y_{Bi}\right)}^{2}\right]}$$

where *n* is the number of wavelengths measured within the spectral range, $y_{Ai}$ is the *i*^th^ sample of the reference spectra, $y_{Ai}$ is the ith sample of the sample spectra, and ${\left|y_{A}\right|}_{avg}$ is the average absolute molar ellipticity of the reference sample. The smoothed spectra corresponding to pH = 7 and T = 75 °C were used as the reference spectra for each protein. A range of wavelengths from 200 to 250 nm were utilized in this calculation. All WSD calculations were completed in R version 4.4.2.

### 2.5. Intrinsic Fluorescence Spectroscopy

Intrinsic fluorescence measurements were conducted on a Jasco-1500 spectrometer using a 10 mm quartz cuvette with protein concentrations of 0.2 mg/mL in 10 mM sodium phosphate buffer containing 100 mM NaCl with pH ranging from 2 to 9 at pH increments of 1 and held at temperatures 75, 80, 85, and 90 °C. Protein samples were excited at a wavelength of 280 nm and emission spectra were recorded at 290 to 450 nm.

### 2.6. 8-Anilino-1-Napthalenesulfonic Acid (ANS) Binding Assays

ANS binding assays were performed using protein concentrations of 0.2 mg/mL. Stock ANS was made such that 2 μL titrations into the protein sample would increase the ANS concentration by 10 μM increments. Fluorescence measurements were conducted after each titration until saturation was reached. All samples were excited at 380 nm and emission was recorded at 500 nm. Measurements were recorded under variable pH buffer conditions (pH 2 and 7) and variable temperature conditions (75, 85, and 90 °C).

### 2.7. Differential Scanning Calorimetry (DSC)

All protein samples were prepared at a concentration of 0.5 mg/mL in 10 mM phosphate buffer. A N-DSC III differential scanning calorimeter was used to determine the melting temperatures (T_m_). Prior to loading, all of the protein samples were subjected to degassing at 25 °C for 15 min. Scans were performed from 25 to 110 °C with a 1 °C/min ramping temperature and at variable pHs (pH 2, 4, and 7). To obtain a stable baseline, buffer runs were conducted before running the protein scans. Blank subtraction was done and data obtained were processed using CpCalc Version 2.2.0.10 software.

### 2.8. Trypsin Digestion

Limited trypsin digestion experiments were performed on all HSP subunits. The initial reaction mixture included 0.2 mg/mL of protein and 0.000781 mg/mL of trypsin in 10 mM sodium phosphate buffer at pH 7.2. Trypsin digestion was carried out at 37 °C and a portion of the reaction mixture was removed at specified time intervals over 15 min. The reaction was arrested by precipitation using trichloroacetic acid. The reaction products were analyzed by 15% SDS-PAGE and the gels were stained using Coomassie Brilliant Blue (Sigma Aldrich, Milwaukee, WI, USA). The band intensities for SDS-PAGE were estimated to measure the percentage of proteolytic digestion using UN-SCAN-IT 6.2 densiometric software. The intensity of HSP samples not subjected to proteolytic digestion was used as a control representing 100% protection from enzymatic degradation.

### 2.9. Structural Modeling

The FASTA sequences of HSPα and HSPβ) from *S. shibatae* strain B12 were uploaded into SWISS-MODEL [48]. Sequences with a similarity of 60% or more were chosen for the templates for both HSPα and HSPβ three-dimensional model generation in PDB format. Select models were subjected to energy minimization and RMSD calculations employing Swiss PDB Deep Viewer 4.1 to select the best model for each protein [49]. HSPα and HSPβ structural data were subjected to scans using EMBL-EBI InterPro 102.0 to visualize the distinct apical, intermediate, and equatorial domains of these proteins [50]. The highly conserved regions of all domains among the different subtypes, including HSPβ-coh, were marked using ESPript 3.0 [51]. The secondary structures for each HSP were visualized using the Swiss PDB Deep Viewer [49]. The NsitePred webserver was used to determine any possible ADP/ATP exchange sites [52]. MODELLER 10.5 [53] was used to generate a homology model of the HSPβ-coh protein. Finally, Visual Molecular Dynamics 1.9.4 (VMD) [54] was used to visualize the three-dimensional structure of each chaperonin.

### 2.10. Molecular Dynamics Simulations

Eighteen different systems were simulated using all-atom unbiased MD with each repeated three times independently for 200 ns. The PDBs generated for HSPα and HSPβ were used to build the initial all-atom models for these HSP subunits. Protonation states of titratable residues were determined at pH 2, pH 4, or pH 6.5 using PROPKA 3.0 [55]. Simulations were performed either at 40, 78 or 88 °C (313 K, 351 K or 361 K, respectively), resulting in eighteen different systems including (1) HSPα-pH: 2-T: 40 °C, (2) HSPα-pH: 2-T: 78 °C, (3) HSPα-pH: 2-T: 88 °C, (4) HSPα-pH: 4-T: 40 °C, (5) HSPα-pH: 4-T: 78 °C, (6) HSPα-pH: 4-T: 88 °C, (7) HSPα-pH: 6.5-T: 40 °C, (8) HSPα-pH: 6.5-T: 78 °C, (9) HSPα-pH: 6.5-T: 88 °C, (10) HSPβ-pH: 2-T: 40 °C, (11) HSPβ-pH: 2-T: 78 °C, (12) HSPβ-pH: 2-T: 88 °C, (13) HSPβ-pH: 4-T: 40 °C, (14) HSPβ-pH: 4-T: 78 °C, (15) HSPβ-pH: 4-T: 88 °C, (16) HSPβ-pH: 6.5-T: 40 °C, (17) HSPβ-pH: 6.5-T: 78 °C, and (18) HSPβ-pH: 6.5-T: 88 °C. 

Each of the 18 systems was built independently for MD simulations using the CHARMM-GUI web server [56]. Each system consisted of one HSP subunit, ∼95,000 TIP3P waters, and 0.15 M NaCl. The size of each system was ∼150 × 150 × 150 Å^3^ and contained ∼318,500 atoms. Each system was first energy-minimized using the conjugate gradient algorithm [57] for 10,000 steps and then relaxed in multiple steps for a total of ∼1.5 ns in an NVT ensemble. Fully unbiased simulations started from the relaxed models in an NPT ensemble, and data were collected every 100 ps for a total of 200 ns in each case. Simulations were conducted using NAMD 2.14 [58] under periodic boundary conditions, and data analysis was conducted using various VMD plugins [54]. All components were modeled using the CHARMM36m all-atom additive force field [59]. A 2 fs time step was used and simulations were carried out at either 313, 351, or 361 K employing a Langevin integrator [60] with a damping coefficient of γ = 1 ps^−1^. Pressure was maintained at 1 atm using the Nosé–Hoover Langevin piston method [61]. The non-bonded interaction cut-off distance was set to 10–12 Å, and the particle mesh Ewald method [62] was employed to compute the long-range electrostatic interactions. For all repeats of each simulation, we started from the initial model built by CHARMM-GUI and independently repeated the procedure from the first repeat. Secondary structural analysis was carried out using the VMD plugin Timeline that uses the STRIDE algorithm [63].

## 3. Results

### 3.1. HSPs Resolved by Sodium Dodecyl Sulfate–Polyacrylamide Gel Electrophoresis

Using sodium dodecyl sulfate–polyacrylamide gel electrophoresis (SDS PAGE) and silver staining, purified HSPα, HSPβ, and HSPβ-coh chaperonin subtypes are resolved (Figure S1). HSPα and HSPβ appear as single bands each at ~60 kDa. This is consistent with prior work [24,43,46]. HSPβ-coh, which is a larger fusion construct, produces a single band at ~73 kDa (Figure S1, middle lane). This higher molecular weight band is expected considering that there is a *Clostridium thermocellum* Type I cohesin domain fused to the core HSP subunit structure [24,43]. The results for HSPα and HSPβ are consistent with prior reports using SDS PAGE and Western blot analysis [24,43,46]. The values are also consistent with the molecular weights derived from the ExPASy ProtParam server [50]. HSPα and HSPβ differ in length by eight residues (560 and 552 amino acids, respectively).

### 3.2. Transmission Electron Microscopy (TEM) Reveals HSP Ring Structures In Vivo

Using purified cell-free HSPα and HSPβ subunits pre-incubated with ATP and Mg^2+^, transmission electron microscopy (TEM) reveals nonameric ring structures of ~20 nm in diameter (Figure S2B). TEM images of cells of *S. solfataricus* strain P2 [9,13] show similar nonameric HSP ring structures [23,24] near the inner membrane surface (see Figure S2A), indicating that the HSP complexes formed in vitro (i.e., cell-free) are also present in viable crenarchaeal cells after thermal shock at 88 °C for 30 min. This is consistent with low-resolution immunogold staining performed in cells of *S. shibatae* strain B12 [64,65], which suggested that HSPs are prevalent within cells of Sulfolobales.

### 3.3. Structural Models Predict Similarities in Three-Dimensional Structures of HSP Subtypes

**Figure 1:**
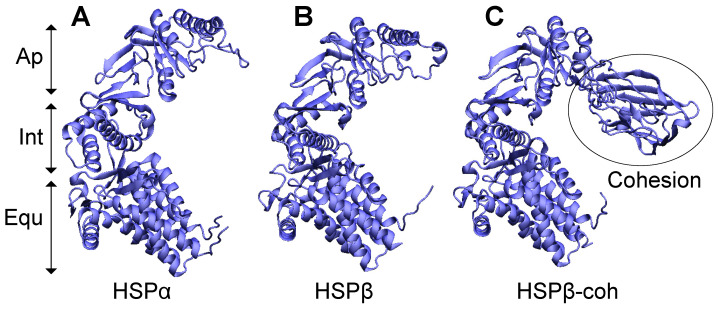
Structural models of natural and engineered HSP subunits. (**A**) 3-D structural model of the HSPα subtype; (**B**) structural model of the HSPβ subtype; (**C**) structural model of the engineered HSPβ-coh fusion construct. The homologous apical (Ap), intermediate (Int), and equatorial (Equ) domains are designated by arrows. The cohesin domain of the HSPβ-coh fusion protein is circled.

Upon resolving the primary structures of each HSP subtype, sequences were used to generate structural models (Figure 1) in a several modeling software packages. Structural models from MODELLER 10.5 [53] for HSPα and HSPβ show similar three-dimensional structures (Figure 1A,B). Structural data were used to compare the secondary structures (i.e., α-helices, β-sheets, loops, and turns), hydrophobic segments, conserved regions, and the tertiary structures of the functional domains (i.e., apical, intermediate, and equatorial) between HSP subtypes. In general, all three HSP subtypes exhibit helix-rich structures with 16 α-helices, 10 β-strands, and a characteristic stem loop between the intermediate and equatorial domains. These data are consistent with other high-resolution structures previously reported [22,23]. For HSPα and HSPβ, ~50% of the amino acid residues are incorporated into α-helices and ~15% form β-strands. Apical domains for HSPα and HSPβ each resolve from a contiguous stretch of residues. However, equatorial and intermediate domains for HSPα and HSPβ form from the convergence of multiple stretches of amino acids and the intermingling of secondary structural elements. Notably, the base structure of HSPβ-coh resembles HSPα and HSPβ but includes the cohesin domain extending from the apex of the core HSPβ permutant (Figure 1C). Since HSPβ-coh is based on the fusion of cohesin to a circular permutant of native HSPβ, the location of residues comprising key secondary structural elements is shifted. Still, the number of α-helices and β-strands and their relative positions in the tertiary structures of the proteins is conserved. Specifically, the HSPβ-coh model has 16 α-helices, 18 β-strands, and the characteristic HSP stem loop. In HSPα, the stem loop resolves at residues 46–58 (KMLIDSFGDVTIT). In HSPβ, the stem loop is located at positions 56–68 (KMFVDSLGDITIT). The HSPβ-coh stem loop resolves at residues 315–327 (KILVDSLGITIT). Despite position shifts for particular amino acids, all HSP subtypes feature very similar three-dimensional conformations.

### 3.4. HSPα, HSPβ, and HSPβ-coh Amino Acid Sequence Alignment Shows Conserved Regions

**Figure 2:**
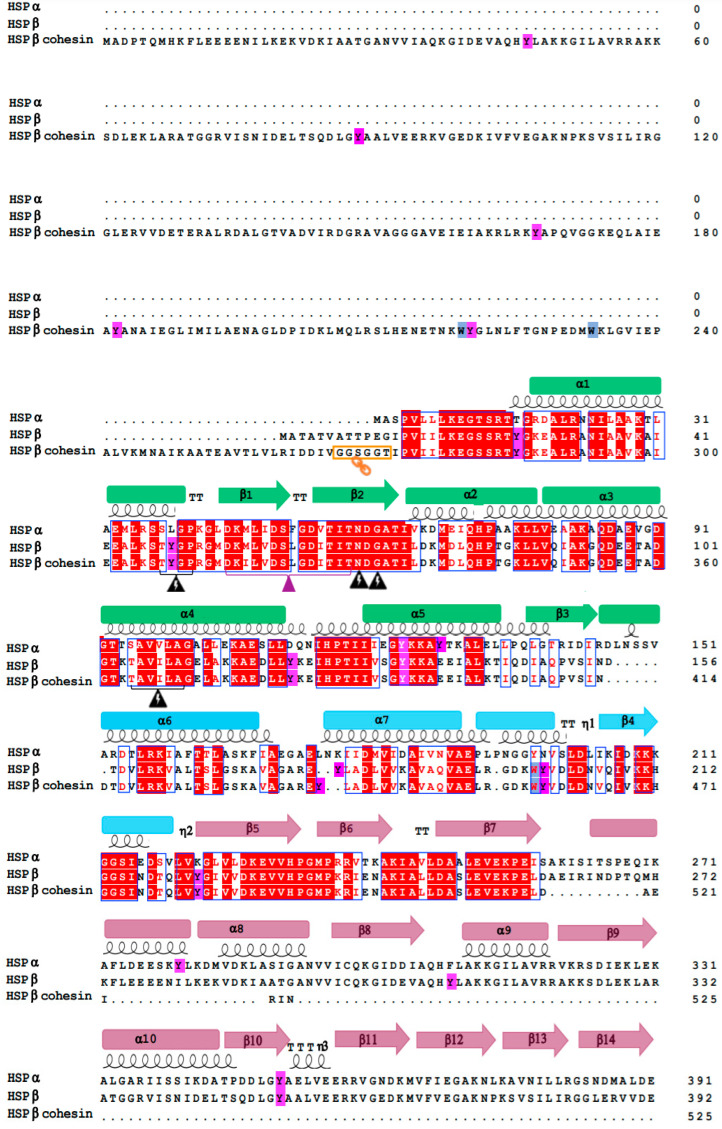

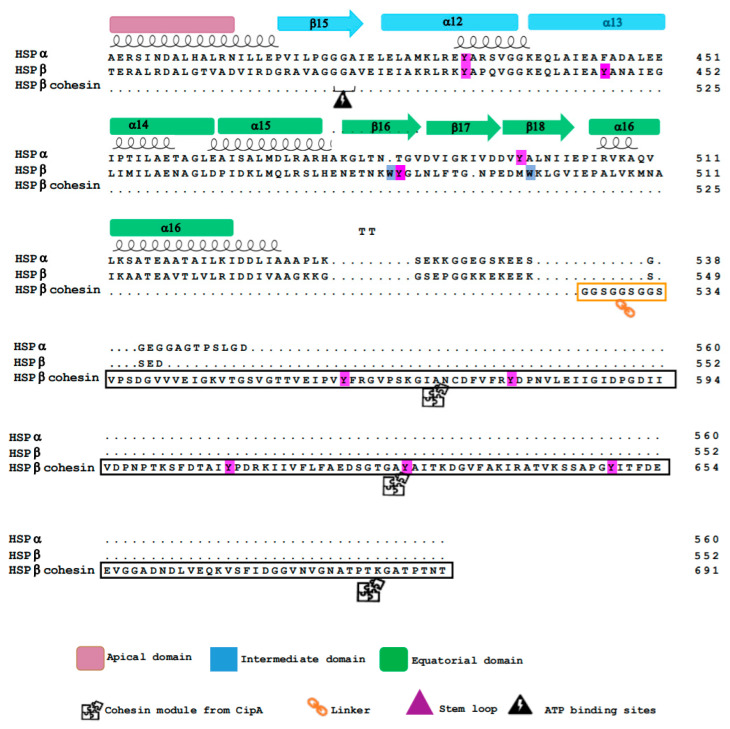
Primary structure alignment of HSPα, HSPβ, and HSPβ-coh. Multiple sequence alignment was generated in ClustalW and visualized by ESPript. The α-helices and β-strands are represented as coils and arrows, respectively. β-turns are noted as TT. Conserved regions are marked in blue boxes and the residues are highlighted in red. The linker at the amino acid position 267 of HSPβ and between HSPβ and the cohesin moiety is marked with an orange box. The domains are color-coded: apical (magenta), intermediate (blue), and equatorial (green). The stem loop region is denoted by a purple triangle. Hydrophobic tyrosine and tryptophan residues are highlighted in purple and blue, respectively. [NCBI/GenBank record locators are: AAA87624.1 HSPα (also known as, TF56), CAA45326.1 HSPβ (also known as, TF55), Q06851 cohesin module of CipA (residues 179–325).

HSP primary structures (i.e., amino acid sequences) were derived from MALDI-TOF mass spectrometry data. Alignment of HSPα, HSPβ, and HSPβ-coh shows conserved runs of amino acids between 57–73, 36–48, and 85–93 for HSPα; 95–103, 46–58, and 67–83 for HSPβ; and 355–362, 316–332, and 325–341 for HSPβ-coh (Figure 2). Sequence alignment also reveals conserved hydrophobic regions and amino acid residues (Figure 2, e.g., blue and purple highlighted residues). For the HSPβ-coh construct, two linkers are present in the primary structure (Figure 2; orange icons). A linker at position 265–270 was engineered to alter the position of the N-terminus and C-terminus in the HSPβ permutant [46]. The second linker at position 526–534 was added to fuse cohesin to the HSPβ permutant.

### 3.5. Differential Scanning Calorimetry Reveals T_m_ for HSPs Under Different pH Conditions

**Figure 3:**
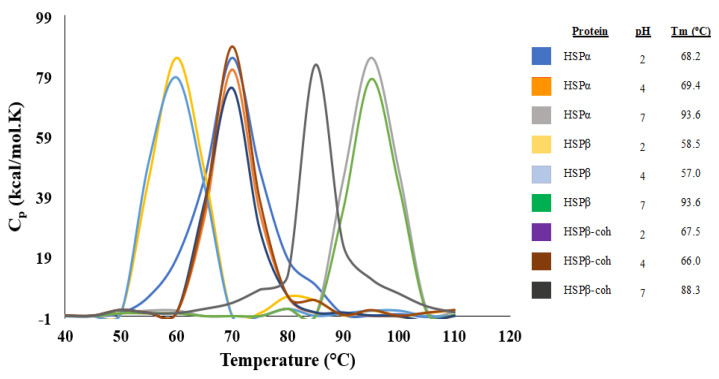
Differential scanning calorimetry (DSC) of HSP subtypes. Analysis of the thermodynamic stabilities of HSPα, HSPβ, and HSPβ-coh monitored by DSC under three different pH conditions: HSPα pH 2 (red), HSPα pH 4 (purple), HSPα pH 7 (orange), HSPβ pH 2 (magenta), HSPβ at pH 4 (green), HSPβ pH 7 (cyan), HSPβ-coh pH 2 (brown), HSPβ-coh pH 4 (blue), and HSPβ-coh pH 7 (black).

Differential scanning calorimetry (DSC) is an analytical tool commonly employed for determining the thermal stability of proteins by measuring changes in heat capacity [66]. DSC was employed to assess the limits of HSP subtype thermal stability. Specifically, HSPα, HSPβ, and HSPβ-coh were subjected to temperatures ranging from 25 to 110 °C. Readings were recorded at 5 °C increments. The melting temperature T_m_ at which 50% of the protein exists in denatured or unfolded states was calculated. T_m_ thresholds for HSPα, HSPβ, and HSPβ-coh were examined under different pH conditions (i.e., pH 2, 4, and 7). At pH 7, T_m_ thresholds for HSPα, HSPβ, and HSPβ-coh were found to be 93.6, 93.6, and 88.3 °C, respectively (Figure 3). The T_m_ for HSPα at pH 2 and 4 shifts from 93.6 to 68.2 and 69.4 °C, respectively (Figure 3). The T_m_ for HSPβ at pH 2 and 4 shifts from 93.6 to 58.5 and 57.0 °C, respectively (Figure 3). The T_m_ for HSPβ-coh at pH 2 and 4 shifts from 88.3 to 67.5 and 66.0 °C, respectively (Figure 3).

### 3.6. Circular Dichroism (CD) Shows Secondary Structure Shifts at Varied pH and Temperature

**Figure 4:**
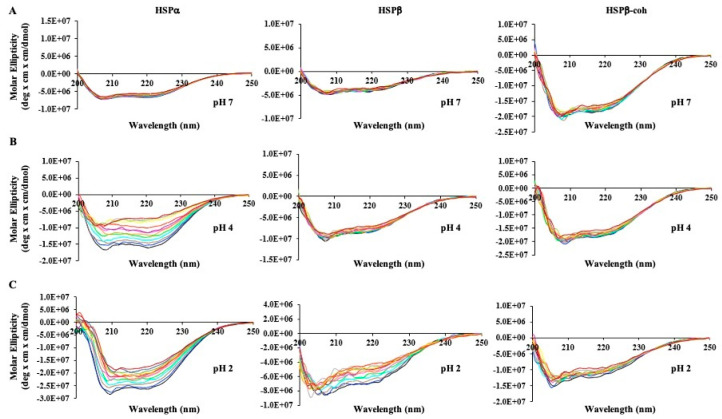
HSP far-UV circular dichroism (CD) spectra. HSPα (**left**), HSPβ (**middle**), and HSPβ-coh (**right**) upon thermal denaturation (25–90 °C) at (**A**) pH 7, (**B**) pH 4, and (**C**) pH 2. Color coding is the same for each spectrum overlay: 25 °C (black), 30 °C (blue), 35 °C (brown), 40 °C (teal), 45 °C (neon green), 50 °C (pink), 55 °C (orange), 60 °C (purple), 65 °C (red), 70 °C (yellow), 75 °C (green), 80 °C (gray), 85 °C (maroon).

To determine the stability of secondary structural elements (e.g., α-helices) under varying conditions of pH and temperature, far-UV circular dichroism (CD) spectroscopy experiments were conducted for each subunit. The CD spectra for each HSP subunit reveal two hypo-elliptical bands around 208 nm and 222 nm (Figure 4), which is characteristic of helical-rich protein backbone structures [67,68].

Each HSP subtype CD spectrum was examined as a function of temperature and pH. At neutral pH 7, varying temperature does not appear to impact the secondary structure of HSPα, HSPβ, or HSPβ-coh (Figure 4A). At pH 7, the thermal denaturation CD spectral overlays of HSPα, HSPβ, and HSPβ-coh superimpose with one another with negligible deviation (Figure 4A). Since HSPs have evolved in thermophilic environments with an intracellular pH typically around 6.5–6.8 [44,45], it is suggested that neutral pH trials at the higher end of the temperature range (75–80 °C) best represent native structural states. The magnitude of molar ellipticity decreases as temperature increases at pH 2 and 4 for HSPα and at pH 2 for HSPβ-coh (Figure 4B,C). Minor spectral shifts were also observed along the x-axis (i.e., wavelength) at pH 2 and 4 for HSPα (Figure 4C).

### 3.7. Weighted Spectral Difference (WSD) Suggests Differences in HSP Subtype Sensitivity to pH

**Figure 5:**
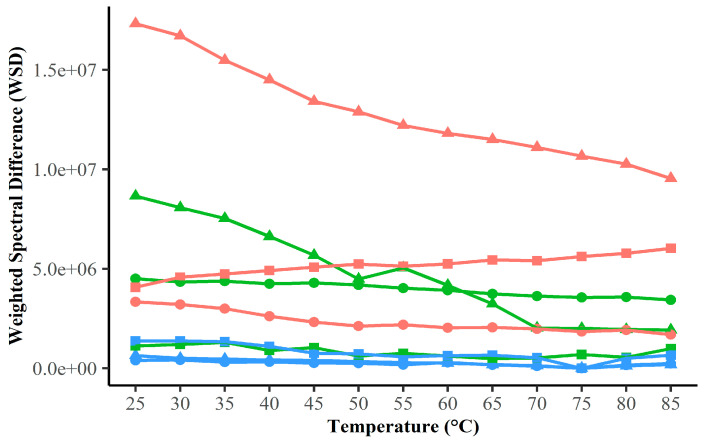
Weighted Spectral Difference (WSD). WSD of Far-UV circular dichroism (CD) spectra for HSPs. HSPα (▲), HSPβ (●) and HSPβ-coh (■) at pH 2 (red), pH 4 (green) and pH 7 (blue). Each point indicates the calculated WSD between the spectra of a given combination of protein, pH and temperature and the spectra of the same protein at pH = 7 and T = 75 °C.

Calculating the Weighted Spectral Difference (WSD) provides a detailed method for quantifying spectral differences in CD data [47]. WSD was employed to provide a more precise comparison of the spectral similarities and dissimilarities between subunits under varying conditions. Again, we consider the spectra at pH 7 and 75 °C to be representative of the native conformation for each HSP subunit. All subunits show similarity across the temperature range of 25–90 °C at pH 7. This is represented by the overlap of WSD values in blue (Figure 5). Interestingly, the thermal denaturation spectra of HSPβ-coh at pH 4 overlap with representative native conformation spectra of all three subtypes at pH 7. However, HSPβ thermal denaturation spectra at pH 4 and 2 exhibit a notable decrease in magnitude. Although the WSD values derived from the thermal denaturation spectra of HSPα deviate the most from the native conformation of HSPα itself and the other subunits at pH 2, all subtypes show dissimilarity when compared to native conformation spectra at pH 2 (Figure 5, red). Analyses of WSD across temperature and pH ranges indicate that changes in pH have a larger effect on the secondary structures of each HSP than changes in temperature (Figure 5). One exception to this trend was seen with HSPβ-coh at pH 4, which closely matched trend curves at pH 7 across the range of temperatures. A negative correlation was seen between temperature and WSD for HSPβ at a low pH. These results suggest that, in terms of secondary structural stability, HSPα is more sensitive to changes in pH and temperature than HSPβ or HSPβ-coh. Despite apparent sensitivity to low pH, these results suggest thermal stability for HSPα, HSPβ, and HSPβ-coh up to 85 °C.

**Figure 6:**
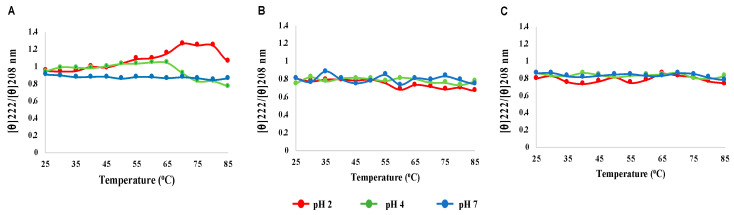
Ratio plots for HSP subunit CD data. Plots of [θ]222/[θ]208 nm as a function of temperature and pH for (**A**) HSPα, (**B**) HSPβ and (**C**) HSPβ-coh.

Although the HSPα CD spectra showed the most significant decrease in magnitude at lower pH, the general spectral profile remained. To determine if these shifts represent significant changes in protein secondary structure, the 222/208 nm ratio was plotted for each HSP subtype spectrum as a function of temperature (by pH condition) for each HSP (Figure 6). The ratio of the molar ellipticities at 222 and 208 nm ([θ]222/[θ]208) is utilized as a criterion to evaluate the presence of coiled-coil helices in proteins [69].

A non-interacting α-helix presents a ratio at 0.9 or below, while ratios of 1.0 or above indicate the presence of coiled-coil helices [70,71]. The effect of varying the temperature and pH of natural and engineered HSP subunits on the [θ]222/[θ]208 ratio was examined to determine if coiled-coil formation occurs due to fluctuations in temperature and pH. The ratio remained unaltered (below 0.9) for all three subunits at pH 7 over temperatures ranging from 25 to 85 °C (Figure 6). There is no overt alteration in the ratio for HSPβ and HSPβ-coh in the lower pH range (2 and 4) (Figure 6). For HSPα, induction of coiled-coil helices appears to take place over the temperature range of 40–85 °C at pH 2 as indicated by a shift in 222/208 nm to 1.0 (and above). The emergence of coiled-coil structures within HSPα at pH 4 appears to take place from 30 to 65 °C (Figure 6A).

### 3.8. Intrinsic Fluorescence (IF) Indicates Tertiary Structure Shifts at Varied pH and Temperature

**Figure 7:**
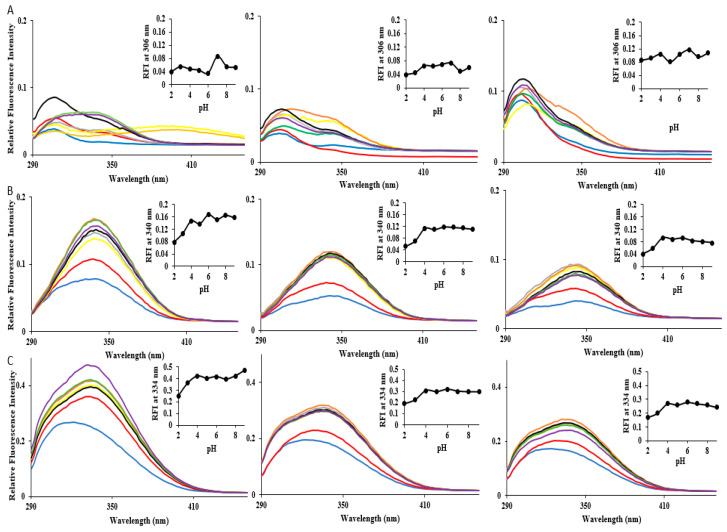
Intrinsic fluorescence spectra of HSPs: (**A**) HSPα; (**B**), HSPβ, and (**C**) HSPβ-coh—were subjected to pH 2–9 at 75 °C (left column), 80 °C (middle column), 90 °C (right column top and middle for HSPα and HSPβ, respectively), and 85 °C (right column bottom for HSPβ-coh). Inset graphs depict the changes in relative fluorescence intensity at the respective native emission maxima for each subunit as a function of pH. Color coding for each graph is as follows: pH 2 (blue), pH 3 (red), pH 4 (gray), pH 5 (yellow), pH 6 (orange), pH 7 (black), pH 8 (green), pH 9 (purple).

Intrinsic protein fluorescence, predominately due to the fluorescent emissions of tryptophan when excited at 280 nm, is a useful method to probe tertiary structural changes in proteins by providing information on stability and folding/unfolding states [72,73]. Tryptophan fluorescence is sensitive to solvent polarity. Tryptophan residue(s) present on the surface of the protein are exposed to solvent polarity, whereas tryptophan residue(s) buried deep within a protein are shielded away from the polar environment. As polarity of the solvent increases, typically, the fluorescence maxima goes through a red shift and the quantum yield decreases [74]. However, in some cases, the tryptophan fluorescence of a protein may be quenched because of quenching by other residues [74]. HSPα does not contain any tryptophan residues. The native conformation shows an emission maximum at 306 nm corresponding to the seven tyrosine residues in the protein located across all three domains: Y280, Y351 (apical); Y198, Y496 (intermediate); and Y124, Y128, Y430 (equatorial). For intrinsic fluorescence readings, HSPα was presented a pH range of 2–9 and held at temperatures 75, 80 and 90 °C (Figure 7A). Intrinsic fluorescence spectra of HSPα, across the pH range, show a maximum emission at ~306 nm, which is consistent with the native conformation of the chaperonin protein (Figure 7A). The environment of the fluorophores for HSPα were unchanged as the emission maxima fluctuated slightly by 2–4 nm with the exception of HSPα at 75 °C and the higher pH range (pH 8 and 9), where it shows emission maxima shift closer to 350 nm. However, the fluorescence spectra of HSPα, under most conditions, exhibit a small hump at 350 nm in addition to the more prominent emission maxima peak at 306 nm. This suggests that a subtle perturbation of the tertiary structure occurs due to temperature and pH fluctuations. 

The relative fluorescence intensity (RFI) is lower at lower pH conditions (pH 2 and 3) across all temperatures (physiological and heat shock). The decrease in intensity could be associated with an increase in compactness (supercoiling of helices), thus resulting in a more buried environment of the fluorophores. This would decrease exposure to solvents, therefore decreasing emissions. Overall, temperature and pH conditions did not lead to significant perturbations in the tertiary structure of HSPα.

HSPβ contains three tryptophan residues and twelve tyrosine residues located across the three domains: Y310, Y352 (apical); W198, Y199, Y223 (intermediate); and Y26, Y49, Y122, Y134, Y431, W433, Y446, Y484, W497 (equatorial). The emission maximum is at 340 nm in neutral pH over the range of temperatures (75, 80, and 90 °C), suggesting that the folded state of the protein is relatively stable and that tryptophan residues for HSPβ are partially accessible to the solvent in its native conformation. No significant changes in emission maxima were observed for HSPβ over this range of pH at 75 °C (Figure 7B), except for at pH 3, wherein the emission maximum decreased to 332 nm. At 80 °C, the emission maximum for all pH conditions except for neutral pH (i.e., pH 7) shifted slightly by 2 nm. At 90 °C, all emission maximum values went through a slight red shift of 2–4 nm. Again, the observed RFI is lower at pH 2 and 3 across all temperatures. Decreases in the emission maxima suggest that the fluorophores may have shifted to a more apolar core perhaps due to an increase in helical content. Like HSPβ, HSPβ-coh only contains three tryptophan residues. No additional tryptophan residues are introduced due to fusion of the cohesin to the HSPβ circular permutant. The emission maximum for HSPβ-coh is ~334 nm in neutral pH over the range of temperatures (75, 80, and 90 °C). Interestingly, at pH 2 and 3, and over the full range of temperatures (75, 80, and 90 °C), HSPβ-coh exhibits a blue shift, as well as a lowered RFI consistent with the other HSPs (Figure 7C).

### 3.9. Anilino Naphthalene 8-Sulfonate Binding Suggests Limited Stability of Tertiary Folds

**Figure 8:**
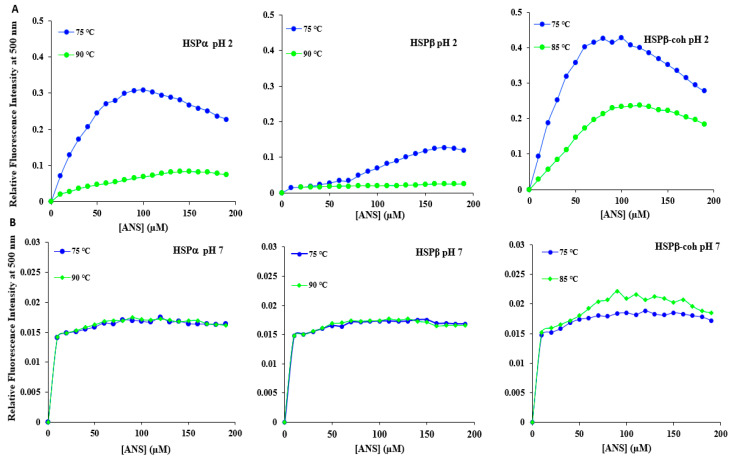
Anilino naphthalene 8-sulfonate binding for each HSP subtype. The ANS binding curves at: (**A**) pH 2 for HSPα (**left**), HSPβ (**middle**) and HSPβ-coh (**right**); (**B**) pH 7 for HSPα (**left**), HSPβ (**middle**) and HSPβ-coh (**right**)—are determined under two temperature conditions 75 °C (blue) and 85 °C (HSPβ-coh, green) or 90 °C (HSPα, HSPβ, green).

Anilino naphthalene 8-sulfonate (ANS) is a non-polar dye which is used to probe the presence or extent of solvent-exposed hydrophobic surfaces in proteins [67,75,76]. This spectrophotometric method is used for studying protein folding in different conditions. The degree of folding, presence of potential intermediate states, or protein denaturation can be monitored by the exposure of hydrophobic residues on the surface of the protein. Differences in the surface hydrophobicity of natural and engineered HSPs appear to be pH-dependent (Figure 8). ANS binding curves for HSPα and HSPβ at pH 7 and 75 °C are comparable (i.e., superimpose). Furthermore, ANS binding for HSPα and HSPβ at pH 7 and 90 °C (i.e., heat shock conditions) exhibits robust congruency. These results suggest that at both physiological pH and under heat shock, the native tertiary folds of these three proteins are not significantly perturbed. ANS for HSPβ-coh upon heat shock (i.e., 85 °C) at pH 7 showed a slight increase in RFI as compared to the binding curve at physiological pH and temperature. For all HSPs at higher temperatures (75, 85, and 90 °C) and neutral pH, the RFI for ANS binding is lower compared to the RFI at pH 2.

### 3.10. Hydropathy Analysis Shows Differences in Hydrophobicity Between HSP Subtypes

The hydropathic indices for each protein were calculated by the method of Kite and Doolittle (1982) and the values were averaged at each equivalent position (Figure S3). Characterization of hydropathy profiles for natural and engineered HSP subunit regions shows that the natural HSPα and HSPβ subtypes were almost mirror images of one another. The mean of the grand average hydropathy score (GRAVY) for natural HSPα, HSPβ and the engineered fusion construct HSPβ-coh was calculated. These GRAVY scores are 0.00625 for HSPα; −0.2 for HSPβ; and −0.11 for HSPβ-coh, respectively. 

Although the hydrophilic profile of these proteins appears conserved across key structural elements, the hydrophobic properties around positions 550–700 of HSPβ-coh were shifted downward (i.e., less hydrophobic) when compared to the natural HSPα and HSPβ subunits.

### 3.11. Trypsin Digestion Assays Suggest a Flexible HSPb Backbone Structure

**Figure 9:**
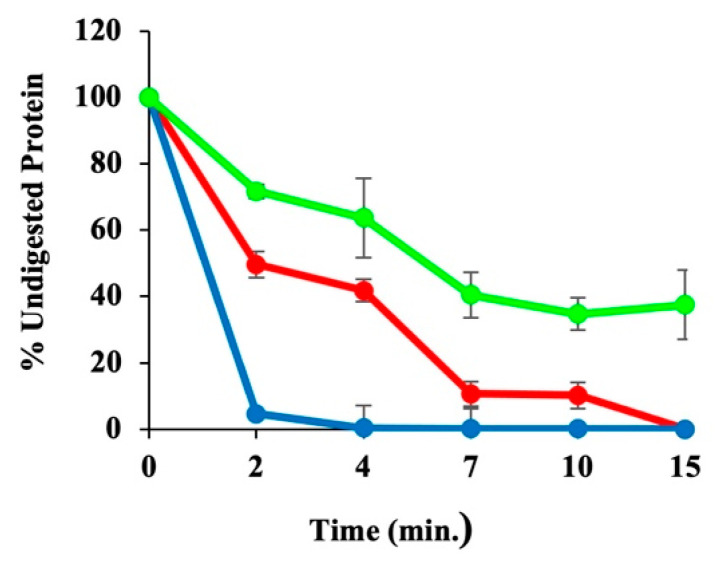
Densitometry for SDS-PAGE gels. Densitometry depicts resistance to limited trypsin digestion for HSPα (red), HSPβ (green), and HSPβ-Coh (blue).

Trypsin, a serine protease, which cleaves proteins at the C-terminal end of lysine and arginine residues, is used to assess the backbone flexibility and provide low-resolution information regarding structural changes in proteins. A time-dependent trypsin digestion was used to compare backbone flexibility between HSP subtypes. The percent digestion post-incubation with trypsin for each HSP was measured by densitometric analysis of the 60 and 73 kDa bands in SDS PAGE gels (Figure S4). HSPβ-coh is readily degraded by trypsin. Specifically, 87% of the protein is digested within 2 min of trypsin treatment. After 15 min of incubation with trypsin (37 °C), the 73 kDa band corresponding to HSPβ-coh fusion protein is completely digested. In comparison, HSPβ and HSPα are digested to 75% and 60%, respectively, after 4 min (Figure 9).

### 3.12. Molecular Dynamics Simulations Reveal Thermostability and pH Dependence of HSPs

All-atom molecular dynamics (MD) simulations are employed to provide a dynamic picture of protein structure at the molecular level. By changing conditions such as pH and temperature, similar to an experimental setting, MD can be use to observe the differential dynamic behavior of proteins under different conditions. Here, all-atom MD simulations with 200 ns simulation time were used to compare the conformational dynamics of HSPα and HSPβ monomers. Given that no direct experimental data exist on the 3D structure of HSPβ-coh, we did not simulate the engineered subunit due to a lack of information for the initial model. However, HSPα and HSPβ were each independently simulated under conditions mimicking pH 2, 4 and 6.5 and temperatures of 40, 78 and 88 °C (9 conditions). For a more reliable comparison between conditions, each subunit was simulated under all nine conditions in triplicate, generating 10.8 microseconds of MD data in aggregate.

**Figure 10:**
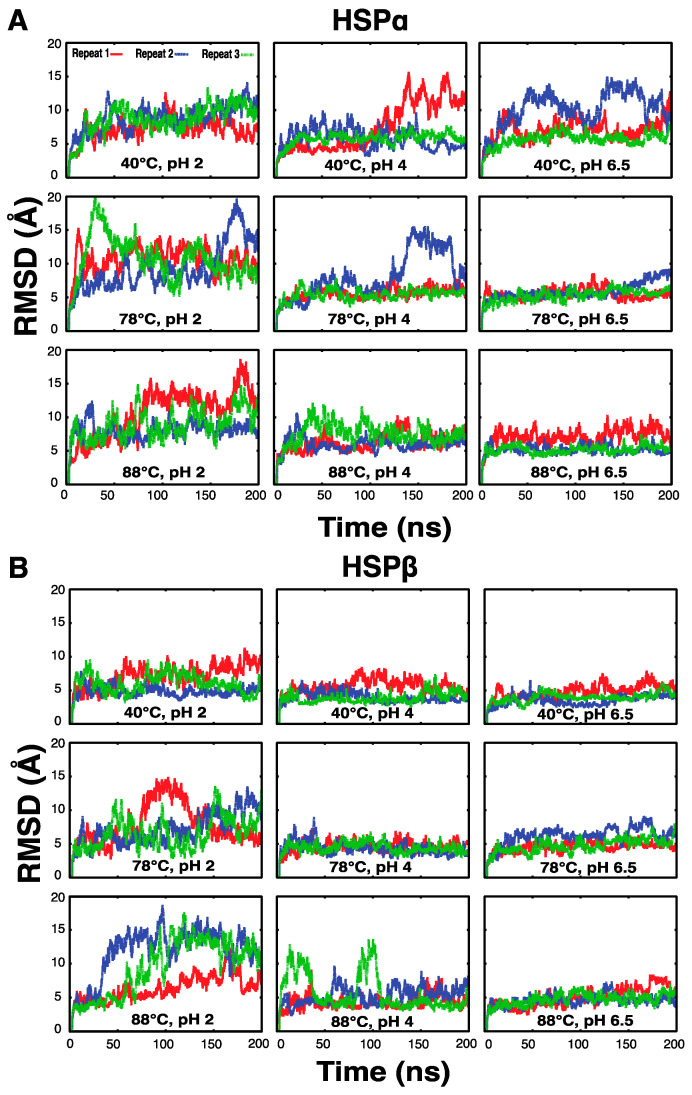
MD simulations of HSPα and HSPβ subtypes. Backbone RMSD time series of triplicate MD simulations of (**A**) HSPα and (**B**) HSPβ -at pH 2, 4 and 6.5 as well as temperatures of 40, 78 and 88 °C with respect to their initial models. (Colors represent individual replicates).

The root-mean-square deviation (RMSD) time series for the initial models of these subunits (see Figure 1) can be used to examine the relative stability of each subunit under different conditions (Figure 10). Simulations show that HSPα undergoes conformational changes within only tens of nanoseconds and almost always shows significant fluctuation and deviation from initial crystal structures when simulated under conditions mimicking pH 2, where RMSD goes beyond 10 Å at some point during the simulation. HSPβ also has signs of instability in cases of acidic pH. Moreover, temperature increase leads to higher instability, a behavior that was not as clearly observed in HSPα under pH 2. In other words, while the pH-dependent behavior of HSPα and HSPβ at acidic pH is clear, the distinction between different temperatures in HSPα is not. The instability of HSPα at the intermediate pH (pH 4) is heightened by decreasing temperature. HSPα in pH 6.5 shows instability only at 40 °C, where RMSD always remains under 10 Å under the higher temperature conditions. On the other hand, HSPβ exhibits stability at pH 4 and 6.5 for all temperature conditions (i.e., 40, 78, and 88 °C) with the exception of one of the replicates at the highest temperature (88 °C) under the intermediate pH condition (pH 4). This again indicates a difference in the behavior of HSPα and HSPβ.

While it seems that HSPα has the highest stability at the highest temperature (88 °C) and pH (6.5), HSPβ is almost stable in both intermediate (pH 4) and high pH (pH 6.5) conditions. If we look at the right side of the HSPα plots (Figure 10A) from bottom to top (high temperature to low temperature) and left to right (high pH to low pH), the instability of this subunit increases in both directions. On the other hand, HSPβ behaves differently: lower temperatures favor stability at least under lower pH conditions. The conformational changes observed in these simulations are not due to secondary structural changes since the secondary structural content of both the HSPα and HSPβ subunits does not show any significant changes during simulation and remains virtually independent of the pH and temperature (Table S1). The helical content (mostly α-helical with a negligible 3_10_ content) varies from 49 to 51% on average for both the HSPα and HSPβ subunits and the β-strand content varies from 15 to 16% for HSPα and from 16 to 18% for HSPβ, with no significant dependence on pH or temperature (Table S1).

## 4. Discussion

Results from this study provide convincing evidence that there are differences in HSP subtype responses (i.e., HSPα versus HSPβ versus HSPβ-coh) to environmental stressors. Despite high structural similarity between these chaperonins, resistance to thermal shock and stability at low, non-physiological pH differs between HSP subtypes. The responses of secondary structural elements versus tertiary structural stability may provide insights into HSP complex function within viable cells under physiological and stress conditions.

### 4.1. Primary Sequence Similarity and High Structural Paralogy Between HSP Subtypes

For cloning HSPα (also known as, TF56) and HSPβ (also known as, TF55) genes from *S. shibatae* strain B12 into a bacterial expression system (i.e., BL21-CodonPlus (DE3)-RIL cells; Agilent, Santa Clara, CA, USA), prior work identified protein bands of ~60 kDa on SDS PAGE [22]. When purified and incubated in vitro with ATP and divalent cations (e.g., Mg^2+^), these HSP60s were shown to form both homomeric and heteromeric octadecameric double-ring complexes [22,23]. Although previous data from an immuno-gold labeled cells suggest expression of HSPs within an archaeal cell [74], there has been little to no evidence that HSP ring structures form in vivo. In this study, we demonstrate through TEM that HSP complexes indeed form within the crenarachaeal cell (Figure S2A).

Prior work demonstrated amino acid sequence similarities for HSP60s derived from select strains of Sulfolobales [22,23]. In this study, we found that HSPα and HSPβ from other species of the *Sulfolobaceae* family also exhibit high sequence similarity (Figure 2). Our data also indicate that sequence similarity between the two natural HSP subtypes yields high structural paralogy (Figure 1). It is this paralogy between HSPα and HSPβ that allows the formation of functional homomeric 18-mer HSP complexes with either subunit in vitro. However, in nature, heteromeric octadecameric HSP complexes are expected [23]. This conservation across species (and genera) underlies the critical role that HSPs play in crenarchaeal survival in the extreme environments that serve as habitats for Sulfolobales. Whether the double nonameric ring complexes consist of two heteromeric ring structures or an HSPα homomeric ring associated with an HSPβ homomeric ring remains unknown. Indeed, stoichiometric favorability of HSPα over HSPβ (or vice versa) in natural systems may vary based on environmental conditions, such as pH, temperature, or other stressors. The extent to which HSPα versus HSPβ gene expression is differentially regulated under cellular stress is the subject of ongoing studies in our lab. Likewise, the extent to which different stressors drive complexation from a pool of residence HSPs is being explored under different conditions of pH, temperature and other stressors (e.g., viral infection).

### 4.2. HSPs Exhibit High Thermal Tolerance but Limited Tolerance to Low pH

Several studies have shown that HSP expression is upregulated during thermal shock [20,22,23]. This supports one of the reported core functions of group II chaperonins—namely, to protect client proteins within the cell under conditions of thermal challenge. Temperature fluxes with the environment will directly impact intracellular temperatures. Therefore, it is reasonable to suggest that HSPs have developed thermal tolerance such that their core structures are stable when other heat-labile client proteins become unstable. Conversely, the crenarchaeal membrane provides a pH gradient between the extracellular environment, which can reach pH < 2 [1,9,13], and the intracellular environment that is maintained at ~6.5 [44,45]. Therefore, it is not presumed that HSPs have evolved tolerance to low pH or the ultra-low pH (e.g., pH < 2) found in some habitats of the Sulfolobales. Still, in these crenarchaea, a significant amount of metabolic energy must be devoted to pumping protons (i.e., H^+^ ions) out of the cell to maintain the cell membrane pH gradient. Under conditions of cellular stress, it is reasonable to suggest that intracellular pH may drop as competition between pathways for metabolic energy ensues. Thus, it is important to understand whether HSPs are resilient and continue to function amidst such pH fluxes.

#### 4.2.1. Natural and Engineered HSPs Exhibit Thermostability at Neutral pH

Results from DSC demonstrate the thermostability of all three HSP subtypes tested at neutral pH (see Figure 3), which is expected of heat shock proteins at physiological pH (i.e., intracellular pH). The natural chaperonins HSPα and HSPβ showed the highest T_m_ values exceeding 93 °C, while the engineered construct HSPβ-coh was lower at T_m_ ~88 °C. Although *C. thermocellum* is thermophilic [77,78], it is not a hyperthermophile. Therefore, cohesion from *C. thermocellum*, which comprises the -coh domain of HSPβ-coh, has not evolved under the same temperature conditions as the group II chaperonins. The lower T_m_ of the HSPβ-coh fusion construct is likely due to the thermal sensitivity of the cohesion domain at these elevated temperatures.

There is a striking drop in T_m_ by ~25 °C with decreased pH for all three HSP subunits, indicating a strong temperature by pH impact. This sensitivity is observed at both low (i.e., pH 4) and ultra-low (i.e., pH 2) pH values. To better understand the nature of this sensitivity, intrinsic fluorescence (IF), trypsin digest, anilino naphthalene 8-sulfonate (ANS), and circular dichroism (CD) were used to parse out relative impacts on tertiary versus secondary structural features.

#### 4.2.2. Integrity of HSP Tertiary Structure Shows pH Dependency

To further explore pH by temperature effects on HSP structural stability, intrinsic tryptophan (Trp) and tyrosine (Tyr) fluorescence was employed. Since the HSPα subtype does not contain Trp residues, IF for HSPα relied on Tyr-based fluorescence. HSPα shows a peak at 306 nm with the highest relative fluorescence intensity (RFI) at pH 7 under all temperature conditions. Although the IF_Tyr_ emission peak does not significantly shift upon altering pH, lower pH conditions result in a decreased RFI (i.e., amplitude) compared to pH 7 across all temperatures (see Figure 7), indicating the possibility of supercoiling, which is substantiated by the 222/208 nm ratio values for HSPα at pH 2 (Figure 7A, insets). In HSPβ, IF_Trp_ shows an emission maximum at 340 nm, indicating partial exposure of Trp residues. Again, there are no notable shifts in the emission spectra. However, there are significant decreases in RFI across all temperature conditions, with the largest drops in magnitude observed at the lower pH conditions (e.g., pH 2). HSPβ-coh has a broader IF spectrum than HSPα and HSPβ, probably due to the additional Tyr residues from cohesin and the shift in the location of Trp residues of the HSPβ permutant. This would account for the peak at ~334 nm and the less prominent shoulder at ~306 nm. A notable blue shift is observed at pH 2 and 3 for HSPβ-coh at all temperatures examined. This suggests that ultra-low pH conditions cause this fusion construct to undergo tertiary structure changes, perhaps leading to an unfolded intermediate state (see Figure 7C). What is clear is that pH does impact tertiary folds in these HSPs.

#### 4.2.3. The HSPβ Backbone Structure Is More Resilient than That of HSPα or HSPβ-coh

DSC indicates a global breakdown in thermostability of HSPs in low pH conditions. IF suggests exposure of internal (i.e., hydrophobic) regions of these proteins at low pH. Both ANS binding assays and trypsin digestion provide further information regarding HSP tertiary structure perturbations under different temperature by pH conditions. At pH 7, ANS shows negligible changes in emissions from non-polar surfaces of HSPα and HSPβ at 75 °C (physiological) and 90 °C (heat stress), suggesting that no significant changes occur with solvent-exposed hydrophobic surfaces in response to thermal stress (see Figure 8). HSPβ-coh at pH 7 shows minor ANS RFI increases even under mild heat shock (i.e., 85 °C). This RFI increase is likely the result of ANS binding to the cohesin domain of HSPβ-coh. ANS binding assays using ultra-low pH (i.e., pH 2) yield notable increases (i.e., 10-fold^+^) in RFI for all three HSP subtypes. This suggests that low pH induces conformational changes that expose non-polar surfaces to ANS binding. Since hydropathy plots indicate that HSPβ-coh features more hydrophobic regions than HSPα and HSPβ (see Figure S3), increases in ANS RFI amplitudes for HSPβ-coh are reasonable.

Limited trypsin digestion (LTD) is another method for assessing protein backbone stability/flexibility. Acknowledging that HSPβ-coh has more trypsin cleavage sites than HSPα or HSPβ with 82, 72, and 75, respectively, LTD shows that HSPβ is the least flexible and thus has the highest backbone stability of all three chaperonin subtypes. HSPα was slightly more susceptible to cleavage than HSPβ, and HSPβ -coh was the most susceptible. Although increased thermostability and increased flexibility can often coincide, generally, thermophilic proteins retain less flexibility than comparable proteins from mesophiles. Although LTD can only be carried out at neutral pH (to avoid disrupting enzyme activity), these data support results from IF, DSC and ANS, suggesting that, overall, HSPβ is the most stable subunit under the pH and temperature conditions tested. This may be due to a more compact three-dimensional structure.

### 4.3. HSP Secondary Structure Exhibits Greater Resilience than Tertiary Structure During Stress

DSC, IF, ANS, and LTD data suggest significant temperature by pH effects on HSP structural integrity; however, these methods focus predominantly on tertiary structure. To explore the resilience of HSP secondary structure to changes in pH and temperature, far-UV circular dichroism (CD) was applied. At physiological pH (intracellular pH ~6.5), all three HSPs exhibit resiliency in the integrity of secondary structural elements over a range of temperatures (25–85 °C). Considered in conjunction with DSC data, CD results suggest that thermal stability is robust for all three subunits at neutral/physiological pH (see Figure 4). Comparisons of far-UV spectral data using a [θ]222/[θ]208 ratio (Figure 6) and Weighted Spectral Differences (WSD) (Figure 5) support the conclusion. Interestingly, the secondary structure appears to be more resilient at pH 4 than pH 2 for all three HSPs. The HSPα secondary structure shows the greatest sensitivity to pH. The apparent stability of secondary structure elements at low pH (pH ~4) with global collapse at ultra-low pH (e.g., pH 2) suggests that the HSP tertiary structure is perturbed, while the secondary structure remains intact under mildly acidic conditions. Whether the conformational shifts shown under mildly acidic conditions represent intermediate conformational states associated with HSP–client protein interactions or HSP complex formation is not known. However, the presence of coiled-coil signatures in CD data suggest the adaptation of a more compact structure at a moderately low pH.

Extensive all-atom MD simulations (10.8 microseconds, in aggregate) reveal that HSPα and HSPβ behave differently under different pH conditions. The pH-dependent behavior of these subunits is mainly attributed to the tertiary structure rather than the secondary structure, within the timescale of our simulations (i.e., 200 ns), as evidenced by RMSD (see Figure 10) and secondary structural (Table S1) analysis of the MD trajectories. This is consistent with the CD experiments, which suggest that the secondary structures of HSP subunits are generally resilient to pH change. The RMSD plots, on the other hand, which are based on the MD simulations, show qualitative similarities in their behavior to those observed experimentally in DSC experiments.

At pH 2, HSPα and HSPβ behave somewhat similarly in that they are fairly unstable. At the highest pH (6.5 computationally and 7.0 experimentally), again, the two subunits behave similarly in that they are both mostly stable. pH 4 is where we see the opposite behavior between the two subunits both computationally (Figure 10) and experimentally (Figure 4) using the CD spectra under different conditions. Computationally, the increase in temperature stabilizes HSPα and destabilizes HSPβ. One interesting aspect observed in the lowest temperature simulations is that the HSPα subunit shows signs of instability while HSPβ remains stable. This may indicate that the initial model of HSPα is associated with a conformation compatible with the high-temperature conformations. Notably, the trend observed in trypsin digestion experiments exhibit consistency and complement the MD simulation data. Specifically, HSPα simulations show more signs of instability when compared to HSPβ, an observation consistent with the higher rate of trypsin digestion for HSPα.

### 4.4. Secondary Structure Stability and Tertiary Structure Flexibility May Impact HSP Function

Structural studies show similarities between the crenarchaeal group II chaperonins (HSPα and HSPβ) and the GroEL superfamily of group I chaperonins in bacteria [79,80]. However, unlike GroEL/ES, which uses a barrel (i.e., GroEL)–cap (i.e., GroES) system to capture client proteins, it has been proposed that the HSP molecular cage may employ a different mechanism. Specifically, it has been proposed that the apical pore of the HSP complex consists of “opened” and “closed” conformations and may transition between states in an iris-like manner [77]. Whether the HSP complex captures clients like the GroEL/ES system or if it opens the apical pore to allow client proteins to access the inner cavity of the molecular cage is not resolved. However, our data suggest that the tertiary structure flexibility is greater under conditions of high temperature and/or low pH, while secondary structures (e.g., α-helices) are more resilient. Shifting of secondary structural elements with respect to one another (i.e., tertiary structure perturbations) would favor an iris-like opening and closing mechanism since greater stability would be required in overall ring conformation to associate two nonameric rings. Alternatively, it may be the case that both mechanisms are possible for HSPα- and HSPβ-containing HSP complexes depending upon the type and magnitude of the environmental stress.

The engineered HSPβ-coh fusion construct serves as an informative comparator since HSPβ-coh complexes are being tested in industrial and agricultural applications. While some structural models suggest that cohesin may interfere with complex formation and pore function suggested for HSPβ- and HSPα-containing complexes (see Figure 1), other models (see Figure S5) show enough flexibility in the linker region to accommodate the positioning of cohesin in multiple locations (e.g., side or back of HSPβ core structure) within three-dimensional space, with comparable energetic favorability. In prior work [24], we showed that complexes containing HSPβ-coh form ring structures similar to HSPβ-only complexes. Thus, complex formation is not disrupted by the cohesin domain. We also demonstrated that complexes containing HSPβ-coh, to which enzymes are bound (i.e., cellulases), enhance enzymatic activity on substrate. The impact of cohesin on apical pore opening/closing requires additional research beyond the scope of this report.

## 5. Conclusions

In conclusion, we have performed an extensive set of biophysical and biochemical analyses of HSP structural stability under different temperature by pH conditions in an effort to determine the relative stability of HSP subtypes: HSPα, HSPβ, and HSPβ-coh. The intention of this work is to better understand group II chaperonin resilience under physical and chemical stressors and relate these stability data to proposed HSP functions. Cumulatively, our data support prior work demonstrating the thermotolerance of the two natural HSP subtypes (i.e., HSPα, HSPβ) and show a significant (albeit slightly reduced) thermotolerance of an engineered HSP fusion construct (i.e., HSPβ-coh).

More specifically, this study tested the extent to which HSP subtypes may exhibit tolerance to low pH. Our data suggest that the HSP tertiary structure is highly susceptible to perturbation at low pH, while the secondary structure is resilient under mildly acidic conditions. The results also indicate that HSPβ is the most resilient of the three tested HSP subtypes under pH x temperature challenge. The resilience (i.e., high stability) of HSP secondary structural elements and the flexibility (i.e., greater instability) of the tertiary structure support the idea that HSP complexes could use an iris-like “open-and-close” mechanism at the apical pore of the 18-mer complex to bind client proteins, especially for natural HSPα- and HSPβ-containing complexes. Since the cohesin domain of HSPβ-coh is attached to the outside apical region of the HSPβ permutant [46], there should be no steric hindrance at surfaces that associate to form ring structures during complex formation. Whether there is notable impact on the open–close kinetics of the apical pore in complexes containing HSPβ-coh is part of ongoing research.

Differences in apical pore kinetics between heteromeric (e.g., HSPβ/HSPα) versus homomeric (e.g., HSPβ-only, HSPα-only) complexes are also currently under study. Whether apical pore open–close kinetics differ between HSP complexes with distinct HSP subunit stoichiometries remains unresolved.

## Data Availability

The original contributions presented in this study are included in the article/Supplementary Materials; further inquiries can be directed to the corresponding author.

## Supplementary Materials

The following supporting information can be downloaded at: https://www.mdpi.com/article/10.3390/microorganisms12112348/s1, Figure S1: Sodium dodecyl sulfate–polyacrylamide gel electrophoresis (SDS PAGE) of HSPs; Figure S2: Transmission electron microscopy (TEM) of HSP complexes; Figure S3: Hydropathy plots for HSP subunits; Figure S4: Limited trypsin digestion of HSPα, HSPβ and HSPβ-coh on SDS-PAGE; Table S1: Secondary structural analysis of MD simulation trajectories.
