# Supplementary material for: Structural Stability Comparisons Between Natural and Engineered Group II Chaperonins: Are Crenarchaeal “Heat Shock” Proteins Also “pH Shock” Resistant?
Source: Microorganisms. 2024 Nov 18;12(11):2348. doi: 10.3390/microorganisms12112348 (PMC11596651; doi:10.3390/microorganisms12112348)
Supplement: Supplementary file 1 [file microorganisms-12-02348-s001.zip › microorganisms-3276822-supplementary.pdf]

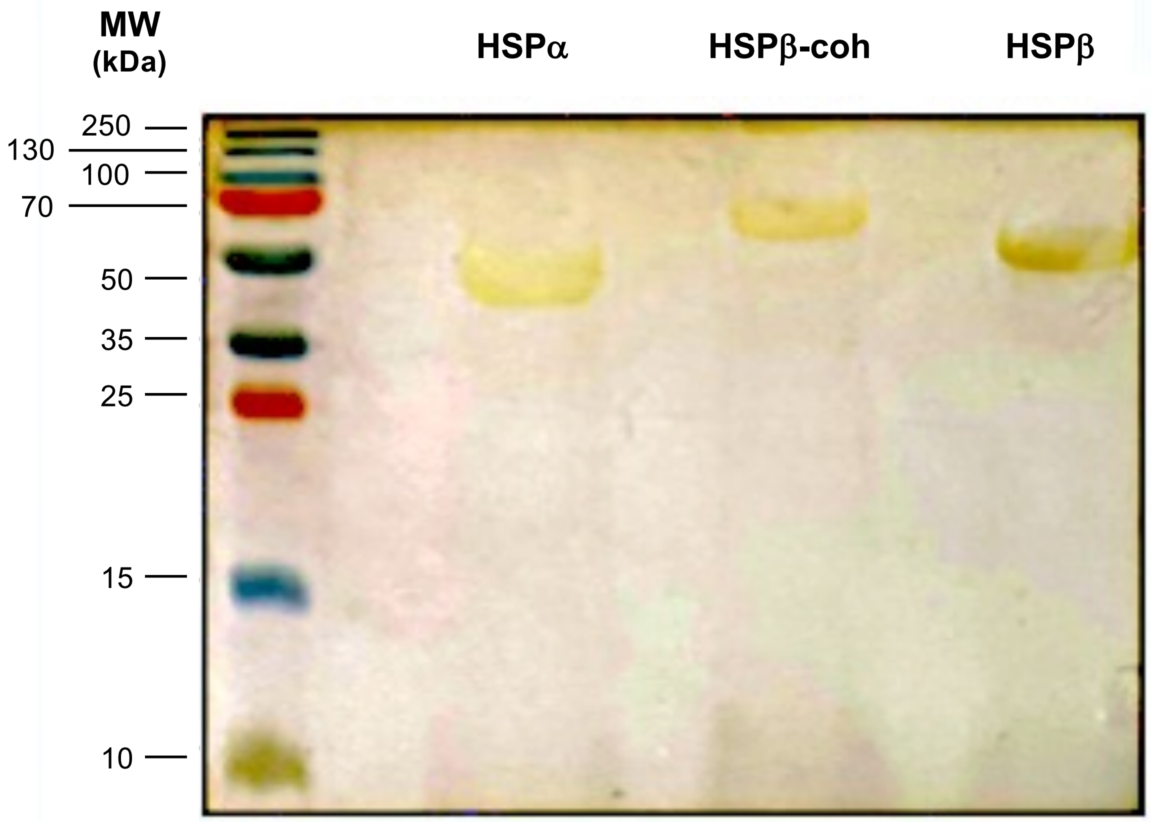

Figure S1. Sodium dodecyl-sulfate polyacrylamide gel electrophoresis (SDS PAGE) of HSPs. SDS PAGE with silver staining resolves two HSP subtypes at ~60kDa: HSPα (left lane) and HSPβ (right lane). An engineered fusion construct, designated as HSPβ-coh, which comprised of HSPβ and cohesin (type I) from *Clostridium thermocellum* is resolved at ~70kDa (middle lane).

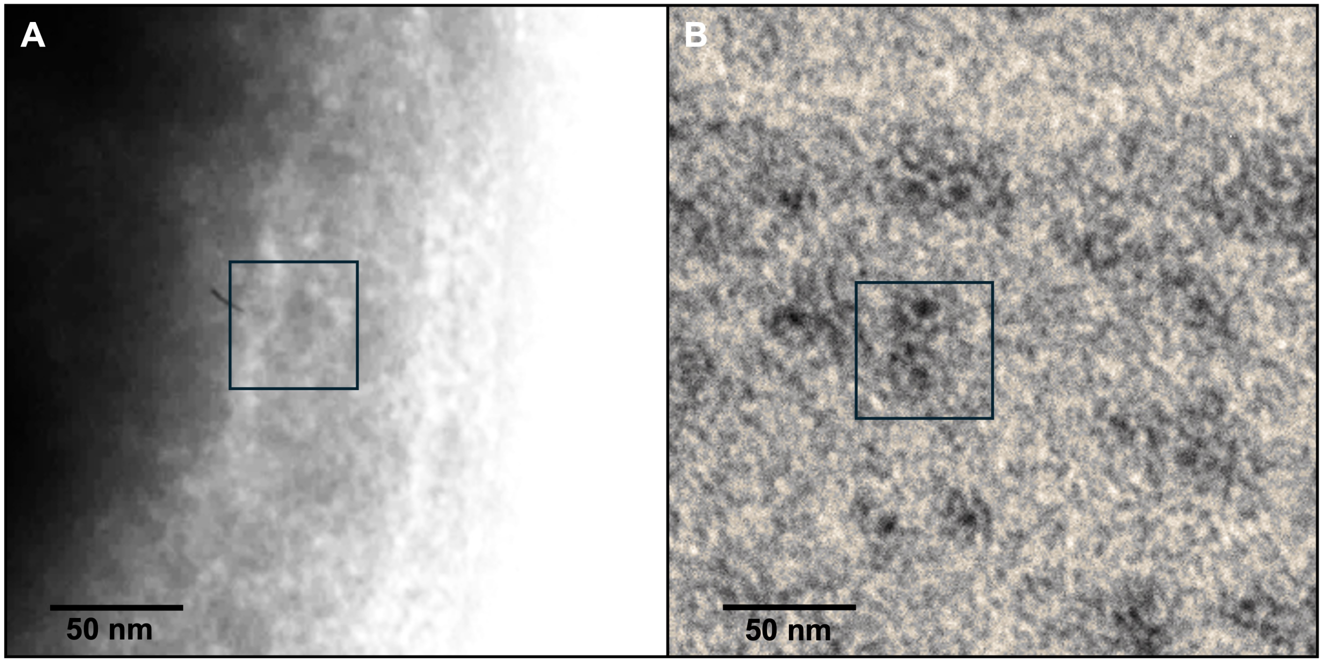

Figure S2. Transmission Electron Microscopy (TEM) of HSP complexes. TEM resolves nonameric ring structures of ~20 nm: (A) within a glutaraldehyde-fixed Sulfolobales cell; and (B) in cell-free in vitro preparations.

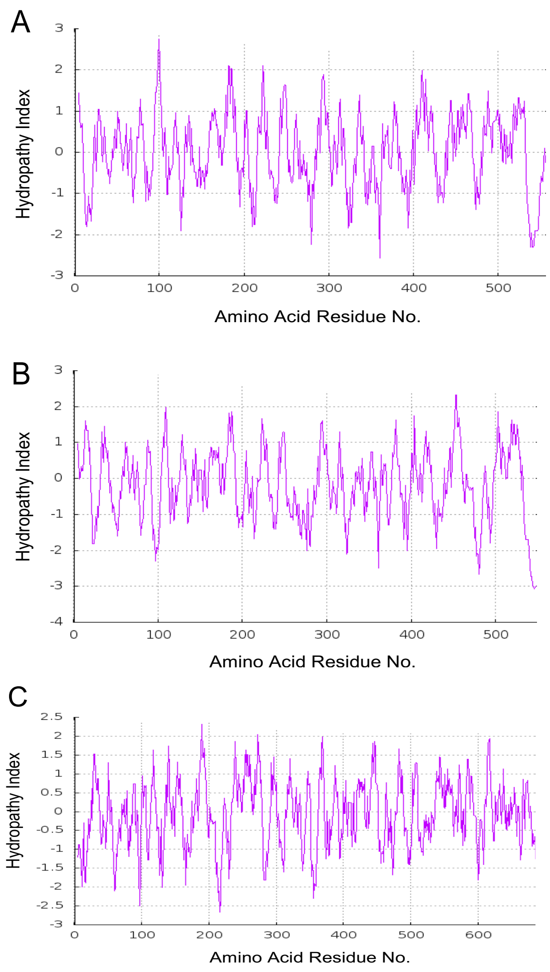

Figure S3. Hydropathy Plots for HSP subunits. Hydropathy indices of (A) HSPα, (B) HSPβ and (C) HSPβ-coh are generated by the by the ProtScale (Expasy) web server using the method of Kyte and Doolittle (1982), with positive (hydrophilic) and negative (hydrophobic) values plotted above and below the center lines, respectively.

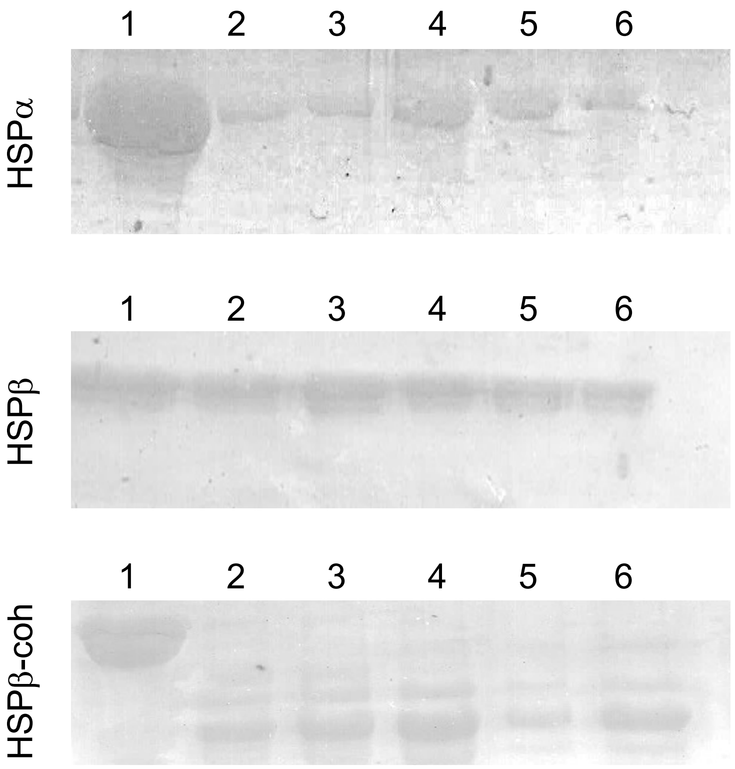

Figure S4. Limited trypsin digestion of HSPα, HSPβ, and HSPβ-coh on SDS-PAGE. Each lane indicates the amount of undigested protein at the respective time intervals. Lane 1= 0 trypsin, Lane 2= 2 minutes incubation with trypsin, Lane 3= 4 minutes incubation with trypsin, Lane 4= 7 minutes incubation with trypsin, Lane 5= 10 minutes incubation with trypsin, Lane 6 = 15 minutes incubation with trypsin.

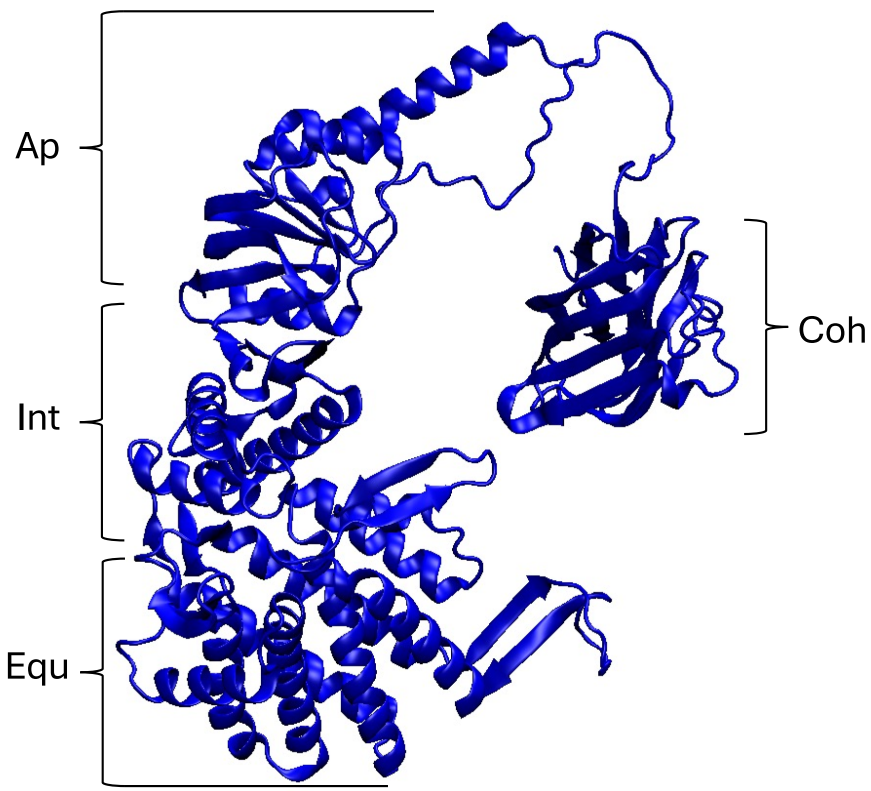

Figure S5. Alpha Fold 3 (AF3) generated HSPβ-coh structure. Structural model shows the core HSPβ apical (Ap), intermediate (Int), and equatorial (equ) domains with the fused C. thermocellum cohesin (Coh) domain anchored from the c-terminus of the HSPβ permutant.

Simulation Setup		Secondary Structural Motif				
**Subunit**	**Condition**	**α-Helix**	**3_{10} Helix**	**β strand**	**Turn**	**Coil**
**HSPα**	**pH 2, 40°C**	47%, 48%	1%, 2%	16%, 16%	14%, 15%	19%, 20%
	**pH 2, 78°C**	47%, 48%	2%, 2%	15%, 16%	15%, 16%	20%, 21%
	**pH 2, 88°C**	48%, 48%	2%, 2%	15%, 16%	14%, 15%	19%, 20%
	**pH 4, 40°C**	48%, 50%	2%, 2%	15%, 16%	13%, 13%	19%, 22%
	**pH 4, 78°C**	48%, 49%	2%, 2%	15%, 16%	13%, 14%	19%, 20%
	**pH 4, 88°C**	49%, 49%	2%, 2%	14%, 16%	14%, 16%	19%, 19%
	**pH 6.5, 40°C**	50%, 50%	2%, 2%	15%, 16%	14%, 14%	18%, 19%
	**pH 6.5, 78°C**	48%, 48%	2%, 3%	15%, 16%	15%, 16%	18%, 19%
	**pH 6.5, 88°C**	47%, 47%	2%, 2%	16%, 16%	16%, 17%	18%, 19%
**HSPβ**	**pH 2, 40°C**	48%, 48%	2%, 3%	17%, 17%	13%, 15%	17%, 18%
	**pH 2, 78°C**	48%, 49%	2%, 2%	17%, 17%	14%, 15%	17%, 18%
	**pH 2, 88°C**	47%, 48%	2%, 3%	16%, 17%	15%, 16%	17%, 18%
	**pH 4, 40°C**	48%, 49%	2%, 3%	17%, 18%	13%, 15%	17%, 19%
	**pH 4, 78°C**	48%, 49%	2%, 2%	17%, 18%	14%, 15%	17%, 18%
	**pH 4, 88°C**	48%, 48%	2%, 2%	17%, 18%	14%, 14%	18%, 18%
	**pH 6.5, 40°C**	48%, 48%	2%, 2%	17%, 17%	14%, 14%	18%, 19%
	**pH 6.5, 78°C**	48%, 48%	2%, 2%	16%, 17%	15%, 16%	17%, 18%
	**pH 6.5, 88°C**	48%, 48%	2%, 3%	17%, 18%	15%, 16%	17%, 17%

Table S1. Secondary structural analysis of MD simulation trajectories. The average secondary structural content is calculated from MD snapshots of each simulation trajectory using VMD plugin Timeline (35). Each row represents one of the 18 unique simulation setups. Each of the two percentages listed for each simulation setup and secondary structural motif is the average secondary structural content from one of the three repeats of the associated MD simulation.
